# PCR for the Diagnosis of Abdominal Angiostrongyliasis in Formalin-Fixed Paraffin-Embedded Human Tissue

**DOI:** 10.1371/journal.pone.0093658

**Published:** 2014-04-04

**Authors:** Rubens Rodriguez, Ana Cristina Aramburú da Silva, Carla Aristonara Müller, Silvana Lunardini Alves, Carlos Graeff-Teixeira, Fernando Fornari

**Affiliations:** 1 Programa de Pós-Graduação: Ciências em Gastroenterologia e Hepatologia, Faculdade de Medicina, Universidade Federal do Rio Grande do Sul, Porto Alegre-RS, Brazil; 2 Faculdade de Medicina, Universidade de Passo Fundo, Passo Fundo-RS, Brazil; 3 Laboratórios de Biologia Parasitária e Parasitologia Molecular, Pontifíca Universidade Católica do Rio Grande do Sul, Porto Alegre-RS, Brazil; The Chinese University of Hong Kong, Hong Kong

## Abstract

To date the diagnosis of abdominal angiostrongyliasis (AA) depends on the histological identification of *Angiostrongylus costaricensis* (AC) in surgical specimens. However, microscopic evaluation is time consuming and often fails in identifying the parasite. We tested whether PCR might help in the diagnosis of AA by identifying parasite DNA in formalin-fixed paraffin-embedded (FFPE) tissue. We used primers based on DNA from *Angiostrongilus cantonensis*. Four groups of FFPE intestinal tissue were tested: (1) confirmed cases (n = 20), in which AC structures were present in the target tissue; (2) presumptive cases (n = 20), containing changes secondary to AC infection in the absence of AC structures; (3) negative controls (n = 3), consisting of normal colonic tissue; and (4) tissue affected by other parasitoses (n = 7), including strongyloidiasis, ascaridiasis, schistosomiasis, and enterobiasis. Most lesions of confirmed cases were located in small and/or large bowel (90%), as compared with presumptive cases, in which 70% of lesions were in appendix (P = 0.0002). When confronted with cases of other parasitoses, PCR showed sensitivity of 55%, specificity of 100% and positive predictive value of 100%. In presumptive cases PCR was positive in 4 (20%). All specimens from negative controls and other parasitoses were negative. In conclusion, the PCR technique showed intermediate sensitivity and optimal specificity, being clinically relevant when positive for abdominal angiostrongyliasis. It allowed a 20% gain in diagnosis of presumptive cases. PCR might help in the diagnosis of abdominal angiostrongyliasis, particularly when the pathologists are not experienced with such disease.

## Introduction

*Angiostrongylus costaricensis* is an intra-arterial nematode harboring wild rodents as a definitive host, and mollusks as intermediate hosts [1]. Human infection is known as abdominal angiostrongyliasis (AA). Several cases have been reported in the Americas [2], particularly in the southernmost State of Brazil [3], [4].

A definitive diagnosis of AA is made through pathological examination of surgical specimens. For histological evaluation it is necessary to include the specimens in paraffin block, in an attempt to find parasitic forms, which demands multiple histological sections and examiner expertise. However, in most cases the finding of parasitic structures such as worms, larvae and eggs is difficult, leading to a presumptive diagnosis or a false negative reading. The presumptive diagnosis is made in the presence of severe eosinophilic infiltration, granulomatous reaction and eosinophilic vasculitis in the absence of parasitic structures [3].

Immunodiagnostic tests in serum have been developed, but show limitations due to cross-reaction with other parasite-related antibodies, such as *Ascaris lumbricoides*, *Strongyloides stercoralis* and *Schistosoma mansoni* [5]. Detection of nucleic acids amplified by polymerase chain reaction (PCR) in serum was recently tested in a small series of patients with AA. In paraffin blocks from animal and human cases, PCR has been shown to be useful for parasite identification, such as *Ascaris* sp [6], *Trypanosoma cruzi* [7], *Neospora caninum* [8], and *Leishmania* spp [9]. However, the utility of PCR for identification of *Angiostrongylus costaricensis* in paraffin blocks from surgical specimens has not been tested [10], [11]. We therefore designed this study to assess whether PCR can identify *A. costaricensis* DNA in samples from infected patients.

## Material and Methods

This study was conducted according with rules of the Helsinki Declaration and was approved by the Ethical Committee of Pontifícia Universidade Católica do Rio Grande do Sul, Brazil (protocol no 10/00174). The referred ethics committee waived the need for consent for utilization of tissue samples.

We used formalin-fixed paraffin-embedded (FFPE) tissue samples from 4 groups of patients archived in our pathology Institute: confirmed cases of AA without other parasitoses (n = 20), presumptive cases of AA (n = 20), negative controls (n = 3), and cases with other parasitic diseases (n = 7). All samples were obtained from an archive of formalin-fixed paraffin-embedded (FFPE) tissue, corresponding to the period between 1996 and 2012. Confirmed cases contained structures of *Angiostrongylus costaricensis* located in intestinal tissue, including worms, eggs and larvae. After identification of a parasitic structure in a standard Hematoxylin and eosin staining (H&E) section, the corresponding paraffin block (5×5×5 mm) was cut with a scalpel. Cases presenting histological findings suggestive of AA, i.e. eosinophilic infiltration, granulomatous reaction and eosinophilic vasculitis, in the absence of parasitic structures were designated presumptive cases and the tissue for PCR was collected in the same manner as the confirmed cases including tissue with the aforementioned reactive changes. Negative controls corresponded to normal colonic tissue obtained from surgical specimens of patients with adenocarcinoma, whereas cases with other parasitoses included patients surgically treated for intestinal infection by *Enterobius vermicularis, Strongyloides stercoralis, Schistosoma mansoni* and *Ascaris lumbricoides*. FFPE tissue from these patients was processed in the same manner as confirmed cases. We also analyzed demographic and clinical features taken from medical records, as well as gross and histological findings. For the latter we graded the eosinophilic infiltrate and granulomatous reaction as mild, moderate and severe.

### PCR

DNA of *Angiostrongylus costaricensis* was extracted from 10 mm thick FFPE tissue. This procedure was repeated three times, generating 3 sub-samples for each case. A number of sections were treated with xylene for deparaffinization and approximately 25 mg of tissue were obtained. This was centrifuged and washed twice with 100% ethanol, and dried at room temperature. DNeasy Tissue kit (Qiagen) was employed for DNA extraction. The primers were designed based on published sequences from mRNA of *Angiostrongylus cantonensis* (Genbank, U17585) [10]. The amplification reaction was performed in a final volume of 25 μL of 0.4 mM of the primer R1 Angio Rev (5′-CTCGGCTTAATCTTTGCGAC-3′) and Angio F1 For (5′ AACGAGCGGCAGTAGAAAAA-3′), using AmpliTaq Gold PCR Master Mix, on the following conditions: 94°C for 4 min, 35 cycles of 94°C for 1 min, 58°C for 2 min and 72°C for 10 minutes in the thermocycler FTC405. For verification of amplicon with the expected product of 232 bp we used the horizontal electrophoresis in agarose gel with ethidium bromide. DNA bands visualization was performed in transluminator. To quantify the DNA extraction we used a Kit Qubit fluorometer, Invitrogen. For the positive control we used 2 worms of Angiostrongylus costaricensis in FFPE, prepared as we did with clinical samples.

### Statistical analysis

We used mean ± SD for quantitative data and frequency and percentage for qualitative ones. Student's *t* test and Fisher exact test were used when appropriate, with the help of Graph Pad Prism version 4. WinPepi software was used to calculate PCR performance. A P-value <0.05 was assumed as indicative of statistical significance.

## Results

### Demographics and clinical characteristics

Most patients with confirmed AA were adults (25th percentile: 21 years), and about two-thirds were men (Table 1). Presumptive cases also occurred in adults, affecting equally men and women. There was no statistical difference in age and gender distribution between confirmed and presumptive cases. Most cases of AA (65%) were diagnosed in the summer and were located mainly in small and/or large bowel. The majority of presumptive cases occurred in the appendix.

**Table 1:** Demographic and clinical characteristics of confirmed (n = 20) and presumptive cases (n = 20) of abdominal angiostrongyliasis.

	Confirmed cases	Presumptive cases	P-value
Age in years (Mean ± SD)	38.8±18.5	36.8±13.4	0.371
Gender M (men) W (women) n (%)	7 W(35)	11 W(55)	0.203
	13 M(65)	9 M(45)	
Season			0.203
Summer	13	9	
Other*	7	11	
Anatomical location			**0.0002**
Small and/or large bowel	18	6	
Appendix	2	14	

*Autumn, winter and spring.

### Pathological findings

Among 20 confirmed cases, 8 compromised the small bowel (5 pseudotumoral and 3 infarction), 6 were localized in the large bowel (all pseudotumoral), 4 compromised ileum and ceccum (all pseudotumoral), and 2 cases presented as acute appendicitis. Perforations were found in the 3 cases with intestinal infarction. Out of 20 presumptive cases, 14 presented as acute appendicitis, 4 compromised the large bowel (all pseudotumoral), and 2 cases with infarction were localized in the small bowel. One of these presented perforation.

**Figure 1:**
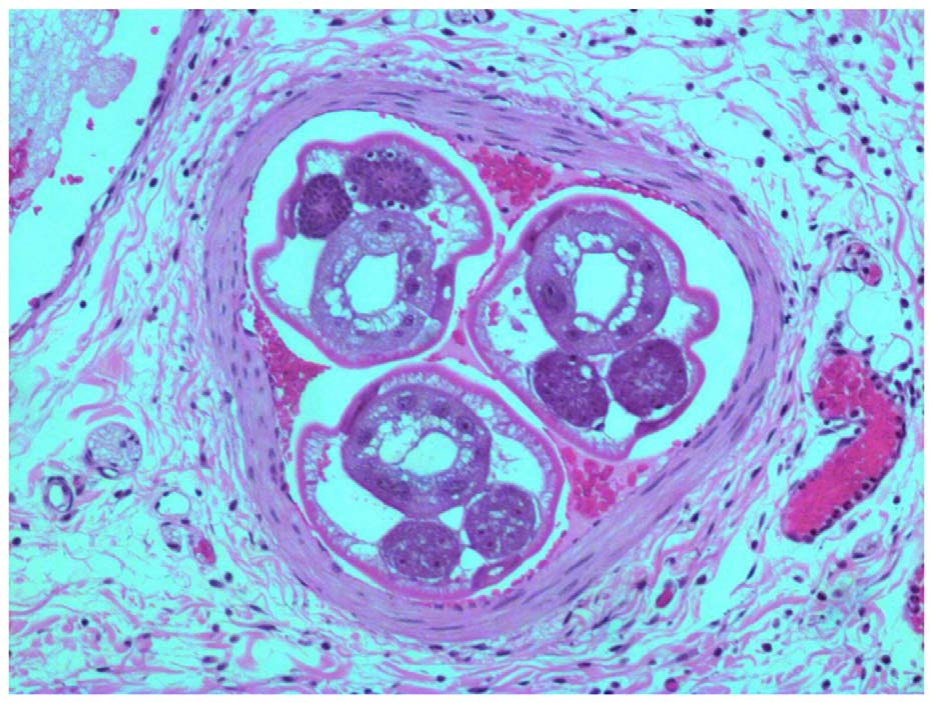
A confirmed case with worms of *Angiostrongylus costaricensis* inside mensenteric artery (H&E, 200x).

**Figure 2:**
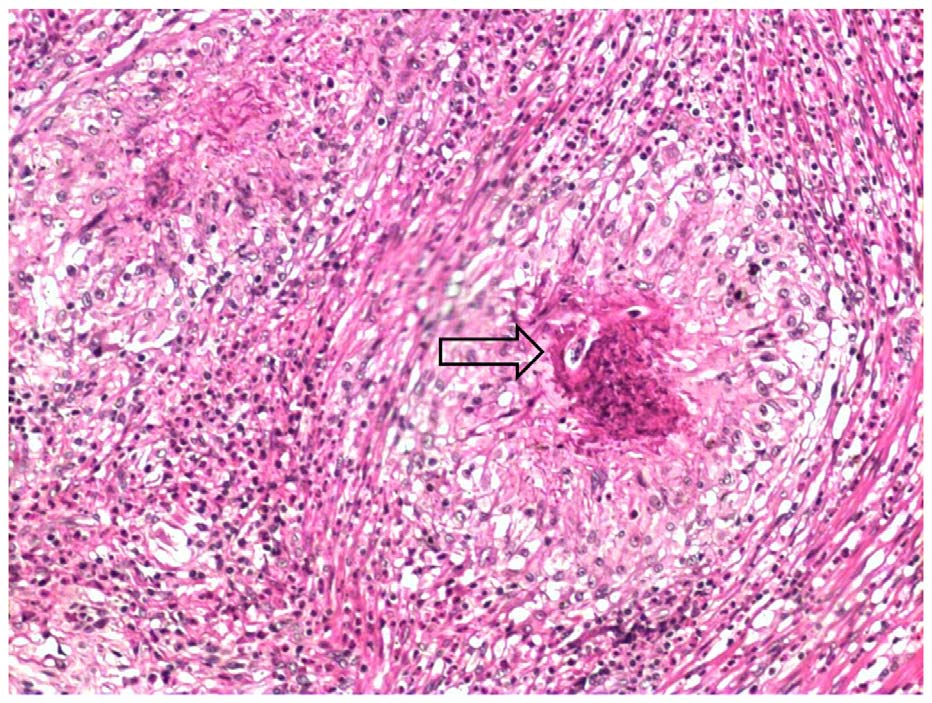
Larvae of *Angiostrongylus costaricensis* (arrow) inside a granuloma in confirmed case (H&E, 100x).

**Figure 3:**
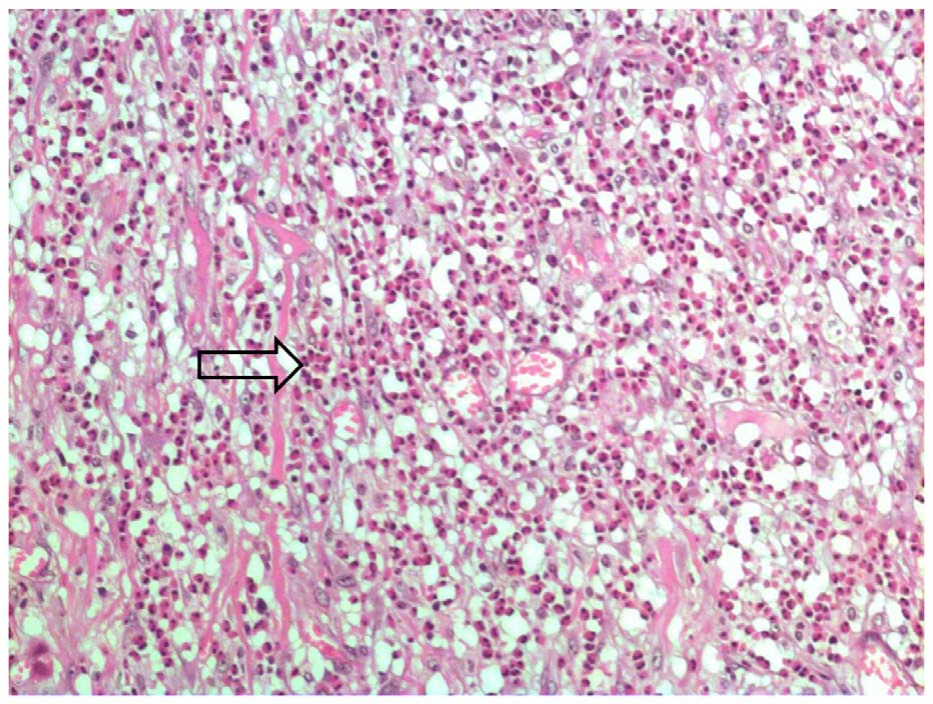
Eosinophilic infiltration (arrow) in bowel wall (H&E, 200x).

Intra-arterial worms were found in 13 of the confirmed cases (Figure 1). The remaining 7 cases presented eggs and/or larvae in the mesenteric arteries (Figure 2). All patients of this group showed eosinophilic artheritis, as well as granulomatous reaction and eosinophilic infiltrates. This latter was severe in 75% of the cases. Eosinophilic arteritis was found in 75% of the presumptive cases, which also presented mild granulomatous reaction and moderate eosinophilic infiltrates (severe in less than 50%) (Figure 3).

### PCR findings

**Figure 4:**
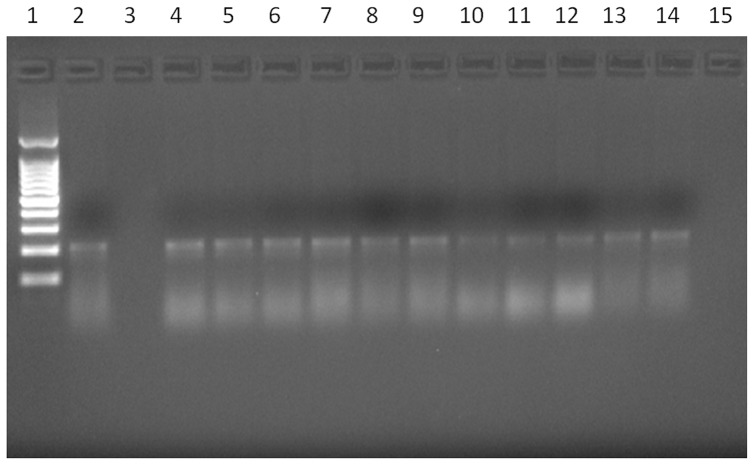
Agarose gel electrophoresis showing PCR positivity of eleven patients with confirmed cases of AA. Lane 1: Molecular weight markers (100-pb ladder). Lane 2: DNA from *Angiostrongylus costaricensis* (positive control). Lane 3: Negative control. Lane 4–14 Confirmed cases with respective DNA concentrations: 12.2 μg/mL; 9.5 μg/mL; 6.5 μg/mL; 11.6 μg/mL; 4.43 μg/mL; 8.88 μg/mL; 1.17 μg/mL; 1.48 μg/mL; 2.28 μg/mL; 2.4 μg/mL; 5.28 μg/mL. Lane 15: Mix (reaction control for detection of possible contaminants).

Amplification of DNA from *A. costaricensis* was positive in areas containing worms, eggs or larvae in a total of 11 (55%) out of 20 confirmed cases (Figure 4): 6 specimens with worms, and 5 containing larvae and/or eggs. Parasite DNA was also seen in FFPE material containing only granulomatous reaction from a confirmed case, in the absence of worms, larvae and eggs. The oldest case with positive PCR was from 1998.

**Figure 5:**
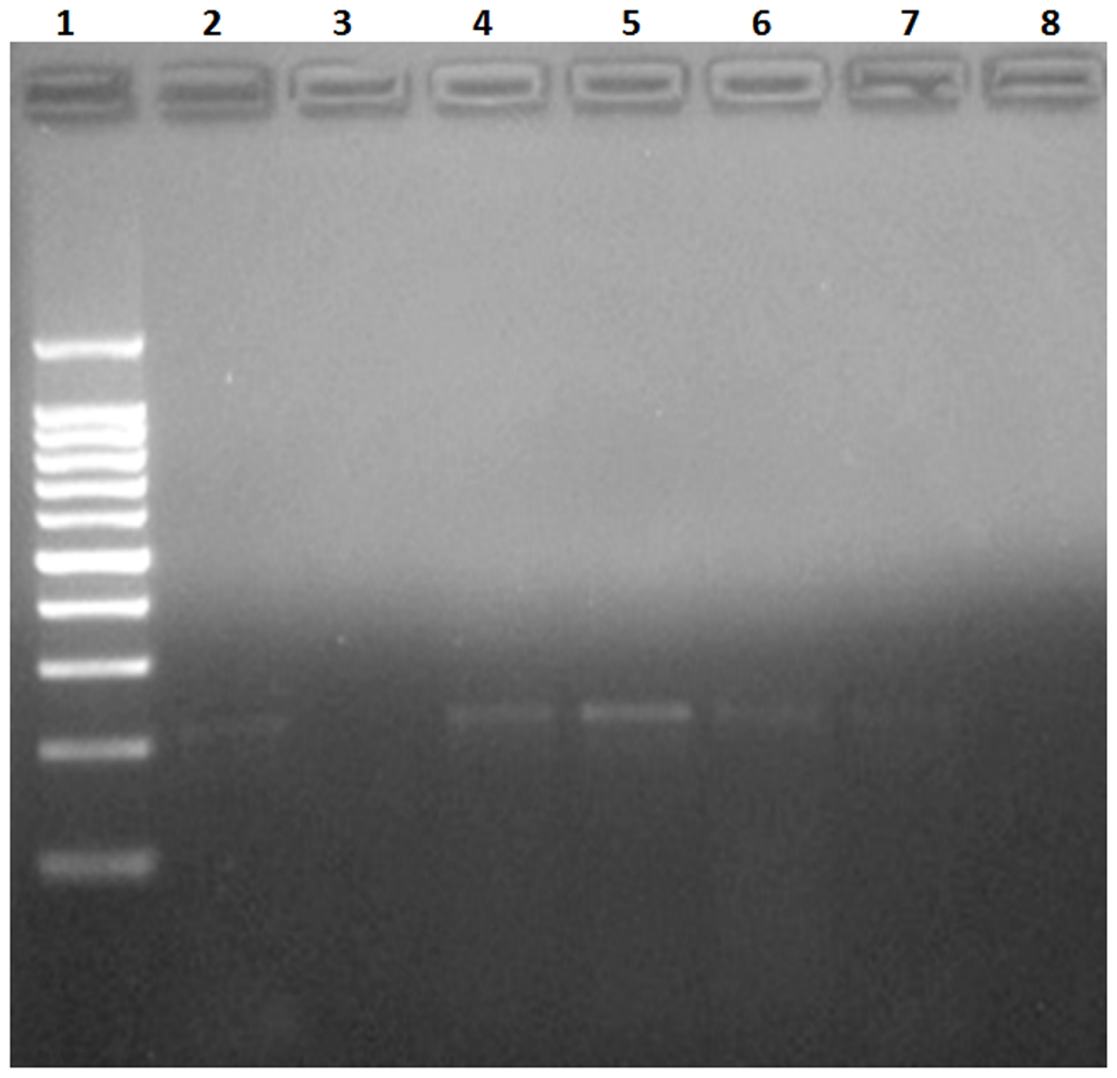
Agarose gel electrophoresis showing PCR positivity of four patients with presumptive cases of AA. Lane 1: Molecular weight markers (100-pb ladder). Lane 2: DNA from *Angiostrongylus costaricensis* (positive control). Lane 3: Negative control. Lanes 4–7: Presumptive cases with respective DNA concentrations: 6.9 μg/mL; 7.52 μg/mL; 2.3 μg/mL; 2.1 μg/mL. Lane 8: Mix (reaction control for detection of possible contaminants).

In presumptive cases, PCR was positive in 4 (20%) out of 20 (Figure 5), in areas containing granulomatous reaction or eosinophilic infiltration. In this group, the oldest case with positive PCR was from 2001. PCR was negative either in control cases or cases with other parasitoses.

When confronted to cases with other parasitoses (Table 2), PCR technique showed intermediate sensitivity (55%), high specificity (100%), high positive predictive value (100%), and intermediate negative predictive value (44%). The rate of true-positive and true-negative (accuracy) was 67%.

**Table 2:** PCR performance in confirmed cases (n = 20) compared to cases with other parasitosis (n = 7).

Test parameter	Performance % (0.95 CI)
Sensitivity	55 (34–74)
Specificity	100 (65–100)
Positive predictive value	100 (72–100)
Negative predictive value	44 (23–66)
Accuracy	67

## Discussion

The diagnosis of AA is often difficult due to the scarce number of parasites in surgical specimens as well as limited pathological expertise. A definitive diagnosis of AA is based on the histopathological identification of parasitic structures such as worms, eggs and larvae in tissue. This process is time-consuming and costly due to the need of a high number of histological sections. When parasitic structures are not found, a presumptive diagnosis of AA may be assumed in cases of suggestive intestinal lesions characterized by a combination of eosinophilic infiltration, granulomatous reaction and eosinophilic vasculitis [3]. However, these features are not pathognomonic of AA and can be seen in conditions such as other parasitoses, Crohn's disease, eosinophilic gastroenteritis and Wegener's granulomatosis [12], [13]. Therefore, new tools designed for parasite identification are necessary to help in the diagnosis of AA.

In this large surgical series of AA, the main findings of our study were: (1) PCR technique using primers of *Angiostrongylus cantonensis* was able to identify genetic material of *Angiostrongylus costaricensis* in paraffin-embedded tissue of patients with AA, including specimens stored for more than 10 years; (2) Although the sensitivity of PCR for detection of the disease in confirmed cases was intermediate (55%), its specificity was optimal (100%), since no case including negative controls and other parasitoses showed a positive test; (3) Despite the absence of parasitic structures in presumptive cases, PCR was positive in 20% of them, decreasing the rate of false negative findings seen with histology alone.

The PCR technique here employed used two DNA sequences of the genus *Angiostrongylus* for confirmation of AA in human cases. Such DNA sequences were designed in our laboratory [10] and were recently tested for identification of *Angiostrongylus* in cerebrospinal fluid of cases with *A. cantonensis* related eosinophilic meningitis [11]. Although these DNA sequences of *Angiostrongylus* were useful in diagnosing cases of either *A. cantonensis* and *A. costaricensis*, urther studies for DNA sequencing of specific *Angiostrongylus* species are still warranted in order to increase diagnostic accuracy of this technique. However, such limitation does not rule out the utility of PCR in the clinical practice, since anatomical and clinical presentation of AA caused by *A. costaricensis* and eosinophilic meningitis related to *A. cantonensis* dictate the final diagnosis. PCR was able to detect parasitic DNA from old FFPE specimens, despite potential tissue degradation inherent to storage time. This finding raises the possibility of using PCR for diagnostic revision of old and unknown cases of archived tissue [14], [15]. Nevertheless, it is not known whether PCR performance would increase when tested in fresh FFPE tissue, as used in the routine of a pathology laboratory. There is no clear explanation for a negative PCR in confirmed cases. A prolonged formalin fixation times could adversely affect the performance of PCR [16], [17].

PCR showed intermediate sensitivity and optimal specificity for detection of *Angiostrongylus* DNA in confirmed cases. In the clinical practice, it means that the test is particularly useful when positive, given its high specificity (low false positive rate) and high positive predictive value. Moreover, in presumptive cases the PCR was able to indicate the diagnosis of AA in 20% of patients. This could be explained by advanced destruction of parasitic structures in retention sites after strong immunologic response [18], precluding identification by standard pathology.

Nevertheless, the pathologist must be aware that a negative result in highly suspicious cases does not rule out that the patient may still be infected. Further studies in prospective cases are needed to confirm the accuracy of PCR in the diagnostic workup of AA.

In our series, confirmed cases of AA occurred mainly in adult men, in contrast with series from Costa Rica, where infants were mostly described [19]. We also observed a concentration of cases presenting during hot seasons, especially in summer. It likely reflects a higher activity of mollusks during hot and wet weather [20], increasing the risk of contamination. Finally, most of our cases compromised small and/or large bowel, particularly with the pseudotumoral form. It is characterized by severe eosinophilic infiltrate in the bowel wall, accompanied by perivascular granulomatous reaction, eventually with retention of parasitic structures such as eggs and larvae [21]. Curiously, cases of perforation were restricted to patients with small bowel lesions, as described by Graeff-Teixeira et al. [3], and few cases of AA presented as acute appendicitis. This contrasts with presumptive cases, in which the appendix dominated the anatomical presentation.

We acknowledge limitations in our study. The primers used for identification of *Angiostrongylus costaricensis* were based on sequences from *Angiostrongylus cantonensis* adult worms. One could question whether these primers would be appropriate for detection of *Angiostrongylus costaricensis* in stages other than adult worms, i.e. eggs and larvae. Nevertheless, we emphasize that primers used in this study were able to identify genetic material of *Angiostrongylus costaricensis* either from adult worms, larvae and eggs in the analysis of human tissue. Future studies using primers properly developed for different stages of *Angiostrongylus costaricensis* are important for confirmation of PCR performance.

In conclusion, we report the molecular identification of *Angiostrongylus* in the largest series of human cases surgically treated for AA. The employed PCR used two DNA sequences of *Angiostrongylus* genus and resulted positive in the analysis of FFPE tissue either for specimens with parasite structures or granulomatous reaction. The successful utilization of PCR here reported points to the need of further studies to establish the diagnostic performance of this technique in a prospective study based on routine pathology.
